# SERS-Based Biosensors for Virus Determination with Oligonucleotides as Recognition Elements

**DOI:** 10.3390/ijms21093373

**Published:** 2020-05-10

**Authors:** Oganes Ambartsumyan, Dmitry Gribanyov, Vladimir Kukushkin, Alexey Kopylov, Elena Zavyalova

**Affiliations:** 1 Mechnikov Research Institute of Vaccines and Sera, Moscow 105064, Russia; ogan-mail@mail.ru; 2 Institute of Solid State Physics RAS, Chernogolovka 142432, Russia; digrib@gmail.com; 3 Chemistry Department, Lomonosov Moscow State University, Moscow 119991, Russia; kopylov.alex@gmail.com

**Keywords:** aptamer, biosensors, nanoparticle, nucleic acids, oligonucleotide, SERS, virus

## Abstract

Viral infections are among the main causes of morbidity and mortality of humans; sensitive and specific diagnostic methods for the rapid identification of viral pathogens are required. Surface-enhanced Raman spectroscopy (SERS) is one of the most promising techniques for routine analysis due to its excellent sensitivity, simple and low-cost instrumentation and minimal required sample preparation. The outstanding sensitivity of SERS is achieved due to tiny nanostructures which must be assembled before or during the analysis. As for specificity, it may be provided using recognition elements. Antibodies, complimentary nucleic acids and aptamers are the most usable recognition elements for virus identification. Here, SERS-based biosensors for virus identification with oligonucleotides as recognition elements are reviewed, and the potential of these biosensors is discussed.

## 1. Introduction

Viral infections are among the main causes of morbidity and mortality of humans, and incur significant financial costs on health care systems. In this regard, it is necessary to develop and apply sensitive and specific diagnostic methods for the rapid identification of viral pathogens.

To date, a lot of methods of virus identification have been widely used; for example, detection via host cell lysis on Petri dishes, amplification-based techniques for specific regions of viral genomes, sequencing of viral genomes, immunoassays for the detection of specific viral proteins, etc. [1]. However, these methods have certain disadvantages that complicate their usage in routine practice in point-of-care diagnostics. For example, some viruses cannot be rapidly cultivated in cell lines [2]. Amplification-based assays, e.g., polymerase chain reactions (PCRs), are a widespread, highly sensitive, efficient and cost-effective technology which makes it possible to amplify target regions of viral genomes [1,3].

With PCR, it is possible to perform quantitative, multiplex analyses, etc. [4,5]. However, PCR is susceptible to contamination [3], and the results can be misleading if incorrect primers are chosen. As a result, PCR requires good laboratory practices and skilled personnel [5,6], which complicates its use in point-of-care diagnostics. Finally, the PCR process requires several hours, so it is also not rapid.

Next generation sequencing is a highly sensitive and specific method that makes it possible to reveal novel genomic sequences and which has a great potential as a clinical method [1,7]. However, at the same time, the application of this technique has been limited because of the high cost of equipment and the requirement of good laboratory practice [1].

Immunoassay methods are based on antigen-antibody interactions, e.g., widely used enzyme-linked immunoassays (ELISAs); they are sensitive and much faster than the methods discussed above, but require high affinity and specific antibodies, especially in the case of multiplex analysis. Despite the relatively low cost of the equipment, most recombinant antibodies are rather expensive, which is the sole weakness of immunoassays in point-of-care diagnostics [8]. Low-cost analogues of antibodies are therefore of high interest for practical implementation.

Nowadays, there is growing interest in the development of biosensors due to their portability, high speed analysis, flexible construction, sensitivity and many others advantages which make them suitable for point-of-care applications [9,10,11,12,13].

## 2. Surface-Enhanced Raman Spectroscopy

Due to its excellent chemical specificity and ability to provide a fingerprint-like spectrum for complex aqueous solutions, Raman spectroscopy (RS) has become one of the most promising optical techniques. RS employs simple and cheap instrumentation, requiring minimal sample preparation. Raman scattering has not found wide application with biological matter due to its inherently weak signal. However, to date, two methods to enhance the sensitivity have been employed, namely, resonance Raman effects, which provides a 10^2^–10^6^ enhancement, and surface-enhanced Raman spectroscopy (SERS), which results in up to 10^8^ enhancement [14,15]. It is widely accepted that the enhancement is due to the coupling of the molecular vibrations of an analyte molecule at the nanostructured surface with locally-amplified electromagnetic fields. These fields, generated at the surface of a metal nanostructure by quasi-particle oscillates, called surface polaritons, form upon interaction of metal surface electrons with photons from the incident light [16]. Noble metals, mostly silver (Ag) and gold (Au), exhibit resonances of surface polaritons in the visible and near-infrared range of the spectrum, correspondingly.

In this review, it is necessary to consider different types of biosensors: colloid nanoparticle solutions (NPs) of different particle morphologies, nanoparticles deposited on the surface, and combined multidimensional materials (e.g., film-over-nanospheres, metal-carbon, metal-polymer and porous nanostructures) [17].

Colloids and nanostructured surfaces have fundamentally different signal amplification mechanisms. In the case of colloid biosensors, the short-range signals are amplified due to the fact that the colloid SERS are determined by localized surface plasmons and ‘hot spots’ arising from interparticle gaps in the nanocluster. The intensity of Raman scattering decreases with an increase of the distance from the ‘hot spot’ as follows, I ~ 1/d^12^, while the SERS signals of the analyte decays to zero at 3 nm from the ‘hot spots’ [18,19].

The SERS effect at the nanostructured surfaces with the interface between the metal and the dielectric layers is not associated with ‘hot spots’ [20]; it is caused by the formation of long-wavelength surface plasmon polaritons focusing of the electromagnetic field near the interface between media with different signs of the real parts of the dielectric permittivity. The intensity of the signals start to decay at distances of 30–60 nm (l) depending on the wavelength of the exciting laser [21] (Equation (1)).

(1) $$l\sim \frac{\lambda_{0}}{2\pi }\frac{\sqrt{\left|\varepsilon_{m}\left(\omega \right)+\varepsilon_{d}\right|}}{\varepsilon_{d}}*0.5$$

where *ε_d_* and *ε_m_* are the real parts of the dielectric constant of the dielectric and metal, respectively, and *λ* is the wavelength of the exciting electromagnetic field.

While the aforementioned techniques for virus detection are sensitive, they are typically time-consuming and expensive. SERS is an outstanding technique in biological applications due to its excellent sensitivity and cheapness. With recent progress in the field, it is now possible to use portable equipment for highly sensitive diagnostics outside the scientific laboratory.

Here, we overview SERS-based biosensors with oligonucleotides as recognition elements for virus identification; these include nucleic acid aptamers (onward-aptamers) and oligonucleotides that are complementary to viral genomes (onward-ASOs, antisense oligonucleotides). Aptamers are considered low-cost analogues of antibodies, so aptamer-based biosensors (onward-aptasensors) are compared with antibody-based biosensors (onward-immunosensors).

## 3. Oligonucleotides as Recognition Elements for SERS

Oligonucleotides are the most promising agents in bimolecular recognition for SERS applications due to their small size and the availability of a wide range of chemical modifications.

Antisense oligonucleotides (ASO) are conventional recognition elements; they are complementary sequences to some unique sites of viral genomes. Analyses require the destruction of viral particles to liberate the genome; and the signal from ASO must be different from the complex between the ASO and the viral genome. ASO production is simple, as it is sufficient to sequence the genomes of target viruses and choose a unique sequence for that particular strain [22,23,24].

Aptamers are oligonucleotides that are capable of recognizing a specific target, e.g., a protein. Aptamers have been widely used in many applications: separation, detection, imaging, diagnostics and therapeutics [25,26,27,28]. Several reviews have been published on aptamers of viral proteins that bind specific viral particles [29,30,31]. The following sections presents examples of oligonucleotide-based sensors for virus detection.

The following advantages make oligonucleotides well suited for SERS applications. They can be chemically synthesized and easily purified, in contrast to most proteins. Aptamers and ASO can be easily modified with a tag, facilitating conjugation with metal- or carbon-based nanostructures that are used for SERS detection [32]. Similarly, a variety of Raman reporter molecules can be conjugated to aptamers and ASO in a site-specific manner; these modifications are available and rather cheap. One more significant feature of aptamers and ASO is their small size (10–20 kDa on average) compared to antibodies (150 kDa for immunoglobulin G); the size matters for SERS, as removal from the surface decreases the SERS signal greatly [21]. The unique properties of aptamers and ASO have enabled the development of various strategies for the detection of biomolecules.

## 4. Direct SERS-Based Techniques for the Identification of Viruses

SERS-based techniques can be divided into two types: direct and indirect. Techniques without reporter molecules (direct or label-free techniques) rely on the identification of the spectrum of an analyte itself. However, direct sensing in biofluids can result in spectra that are difficult to interpret due to the different and unpredictable enhancement of components [33], and due to overlapping of the spectral bands, which makes it difficult to discriminate the target [34]. Regardless of the restrictions, direct SERS biosensing has found some uses in the identification of the characteristic spectra of serum from patients infected with hepatitis B compared to healthy people [35]. The measured spectra of samples from patients with the hepatitis B virus differed from those in samples from healthy people. Principal component analysis and linear discrimination analysis were used to differentiate the spectral data. The differences in spectra arise from an increase in the L-arginine peak, lines of saccharides, phenylalanine, tyrosine, as well as from a decrease in the proportion of nucleic acid, valine and hypoxanthine in the serum of patients with hepatitis B. Diagnostic sensitivity and specificity were 91.4% and 83%, respectively. In this particular example, the time of analysis was one of the main advantages; in the case of PCR, it was about 4 h, and for SERS, about 10 min for each sample.

The development of statistics methods of spectra analyses has allowed us to distinguish among the SERS spectra of different strains of the same virus. For example, silver nanorod arrays were used as substrates for the SERS-based detection of several pathogenic viruses such as the adenovirus, rhinovirus, and human immunodeficiency virus [36]. Additionally, it is possible to distinguish between different strains of the influenza A virus [37], respiratory syncytial virus [38] and rotavirus [39]. More recently, a novel, multilayered gold SERS-substrate [40] was fabricated to identify adenovirus and coxsackievirus at concentration levels of 10^6^ pfu/mL (where pfu is plaque forming unit, i.e., the number of infectious viral particles). The application of modern statistical analytical methods, such as principal component analysis (PCA), facilitates the classification of viruses based solely on their intrinsic spectra. Overall, the technique reached >98% sensitivity and 100% specificity for the measles virus. Different virus strains were readily identified [41].

**Figure 1:**
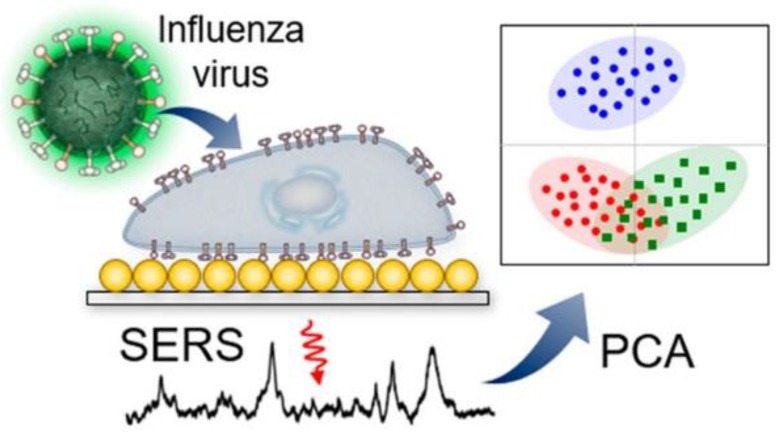
Label-free identification of cells infected with different influenza viruses based on SERS with further principal component analysis (PCA). Reproduced with permission from [43]. Copyright American Chemical Society, 2019.

Au/Ag multilayered nanorod arrays have been used for the detection of influenza A virus strains H1N1, H2N2 and H3N2. These strains were distinguished at concentration of 10^6^ pfu/mL [42]. Similar results were obtained for infected cells by Lim et al. [43]. The basic principle is illustrated in Figure 1.

Nucleic acid aptamers were used as the primary recognition elements in label-free techniques. For example, substrates with silver nanorods with immobilized polyvalent aptamers that are capable of binding the envelope protein of influenza virus were used to detect several influenza virus strains, namely A/Uruguay/716/2007 NYMC X-175C, B/Brisbane/60/2008, A/Brisbane/59/2007 IVR-148 [44,45]. All these influenza strains were detected, whereas respiratory syncytial virus was not captured, being a negative control. Although the experiment was carried out in a buffer and not in a biological fluid like whole blood or serum, the specificity of the biosensor was demonstrated.

These initial results demonstrated that SERS could be used in combination with multivariate statistical methods for the rapid identification and classification of viruses. The results suggested that SERS permits rapid and accurate virus identification, including differentiation of a single pathogen at the strain level. One of the biggest disadvantages of SERS-based techniques is the inability to determine virus titer, because of the nonlinear SERS enhancement at high analyte concentrations and nonuniform adsorption of molecules on the nanoparticle surface, as well as the formation of irregular ‘hot spots’ that decreases signal intensity.

## 5. Indirect SERS-Based Techniques for the Identification of Viruses

Indirect SERS-based techniques could be applied as an analytical tool for the quantitative identification of a target substance. Specificity is provided by molecular recognition elements such as antibodies, aptamers or other specific binding molecules immobilized on the surface of sensors which selectively capture analyte molecules, placing them close to the ‘hot spots’ on the surface.

The sandwich-like construction of biosensors is used in indirect SERS biosensors. The SERS signal comes from a reporter molecule, not from the analyte itself. The Raman reporter molecules should be water soluble, easily conjugated or intercalated to oligonucleotides; in addition, narrow vibrational Raman bands, high photostability, and minimal autofluorescence are preferable. Detailed information on the synthesis of SERS reporter molecules, enhancing photostability and methods of conjugation may be found in several reviews [46,47,48,49].

### 5.1. ASO-Modified Colloid Nanoparticles for the Detection of Viral Genomes

A particularly convenient feature of colloid-based biosensors is that analysis may be performed in a one-pot fashion. Au and Ag nanoparticles are preferable because of their stability and remarkable plasmonic properties. Notably, the aggregation of nanoparticles could be used to detect viruses [50,51]. In these systems, viruses can be detected by visual observation or the colorimetric method.

In addition to color change, the aggregation of nanoparticles generates plasmonic coupling between nanoparticles and, as a result, the SERS effect. Most studies with colloid nanoparticles (NP) and oligonucleotides as recognition elements are connected with the detection of viral nucleic acids. In the study of Hu et al., two types of Au NP were used. The first NP was ASO-modified; it bound to the substrate in the presence of ASO, and the second NP bound to the first, producing dendrimer-like structures [52]. These two complementary NP acted as bricks to build up the multiconstruction with a lot of ‘hot spots’; additionally, the whole construction was attached to the surface. The Raman signal was significantly enhanced; as a result, the human immunodeficiency virus genome was detected at concentrations as low as 10^−19^ M (10^−23^ mole per probe), with the ability to distinguish a single base mismatch.

Gene mismatches in the H1N1 influenza virus were detected using colloid Au NP nanoparticles and ASO labeled with fluorescent dye [53]. The signals from the ASO, a partially complementary duplex with one mismatch and a completely complementary duplex, were different. The assay is useful for fast discrimination of highly pathogenic strains from seasonal influenza strains.

**Figure 2:**
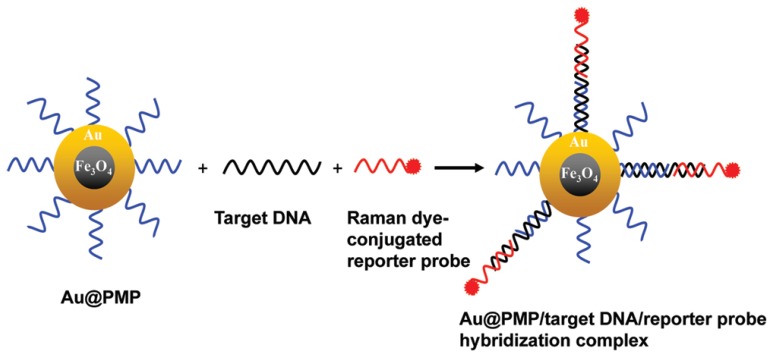
ASO-based identification of viral genomes in nanoparticle solution. High specificity is achieved due to a ternary complex with ASO labeled with a Raman-active compound. Reproduced with permission from [54]. Copyright American Chemical Society, 2012.

A magnetic capture-based SERS assay for viral genome detection was developed using Au-coated, paramagnetic NPs that are useful both as SERS substrates and as an effective strategy to target viral genome purification from other components [54]. One ASO is connected to a NP, another is conjugated with a Raman dye; efficient SERS is possible only for the ternary complex that is assembled on the viral genome (Figure 2). Genomes of the Rift Valley Fever or West Nile viruses were determined with a limit of detection in the range of 20–100 nM.

### 5.2. ASO-Modified Nanostructured Surfaces for the Detection of Viral Genomes

A variety of substrates have been used for virus detection. Substrate-based detection has many advantages, including simplicity, low cost and miniaturization. Several examples with ASO as recognition elements are discussed further.

**Figure 3:**
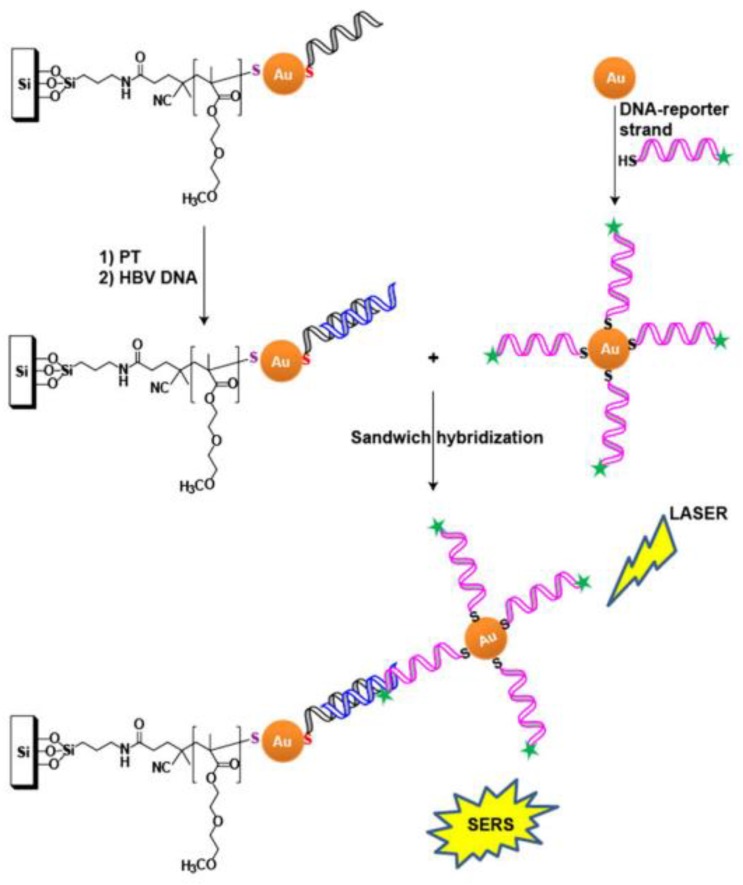
ASO-based identification of viral genome on solid substrates. Ternary complex formation is necessary for the generation of a SERS signal. Viral genome is shown in blue, and primary and secondary ASO are shown in black and magenta, respectively. Reproduced with permission from [55]. Copyright Wiley, 2017.

ASO-modified, nanostructured surfaces were used to detect the genome of the hepatitis B virus [55]. ASO were immobilized on Au NPs that were deposited on a silicon substrate. The second ASO was labeled with Raman dye and immobilized on colloid Au NPs. Similar to the previous assay, efficient SERS is possible only for the ternary complex that is assembled on the viral genome (Figure 3). It was shown that an increase of temperature induces the SERS signal, due to increased aggregation of NPs. The detection limit was ~0.44 fM for 25 °C and ~0.14 fM for 37 °C; the duration of the analysis was about 4 h.

One more biosensor with a similar setup was developed to detect the genome of the hepatitis B virus [56]. Ag NPs coated with silicon dioxide labeled with Malachite Green dye were used as SERS probes. The substrate and nanoprobes were functionalized with ASO. The signal was obtained from a ternary complex, and the detection limit was 50 aM.

The Mirkin group reported the detection of various virus genomes including hepatitis A, hepatitis B, human immunodeficiency, Ebola, and Variola [57]. The assay is similar to the that in the previous work. The main difference is in the Ag shell of the NPs that was used to produce strong SERS signals; the limit of detection was 20 fM. The multiplexed detection of various viruses was successfully accomplished using different Raman molecules for different viruses.

The occurrence of false positives and false negatives is an important factor affecting further applications of biosensors. Therefore, a dual control system is useful with simultaneous measurement of fluorescence and SERS [58]. The genome of the respiratory syncytial virus was detected using a hairpin ASO labeled with a fluorescent dye on one end and immobilized on a substrate via the opposite end. In the absence of the target sequence, the Raman signal was recorded. When the target genomic nucleic acid was hybridized, the Raman dye was shifted away from the surface; the Raman signal was decreased, whereas fluorescence signal was strong. Thus, the inverse dependence of the Raman signal and fluorescence provided a double control for the biosensor.

Similar techniques were developed to detect the genome of the avian influenza virus H5N1 [59,60]. The signal had a linear dependence on the target RNA within the range of 0–60 attomoles, and the detection limit was 2.67 attomoles (the quantities were calculated for the volume irradiated with a laser beam). The technique was able to distinguish between similar sequences with single mutations.

Raman dyes can be introduced through intercalating compounds that make a complex with double-stranded DNA during the assay setup, instead of chemical modification of ASO alone. The biosensor for the detection of the Epstein-Barr viral genome in blood plasma was made using this principle [61]. The rhenium carbonyl was used as a Raman reporter; it was bound to intercalating dye daunorubicin to label the duplex between the viral genome and ASO. In this case, biological objects had no signals in the region of 1780–2200 cm^−1^; therefore, the spectral lines of metal carbonyls did not overlap with lines of other objects [62,63]. Thus, the authors consider it possible to use this method to detect target DNA in biological fluids.

### 5.3. Aptamer-Modified Nanostructured Surfaces for Virus Detection

ASO are intended to capture genome fragments. In contrast, nucleic acid aptamers are capable of capturing viral proteins. The aptamers were successfully used in indirect techniques as primary and secondary recognition elements due to the possibility of extensive modification of oligonucleotides. Very few studies have been done in this field.

**Figure 4:**
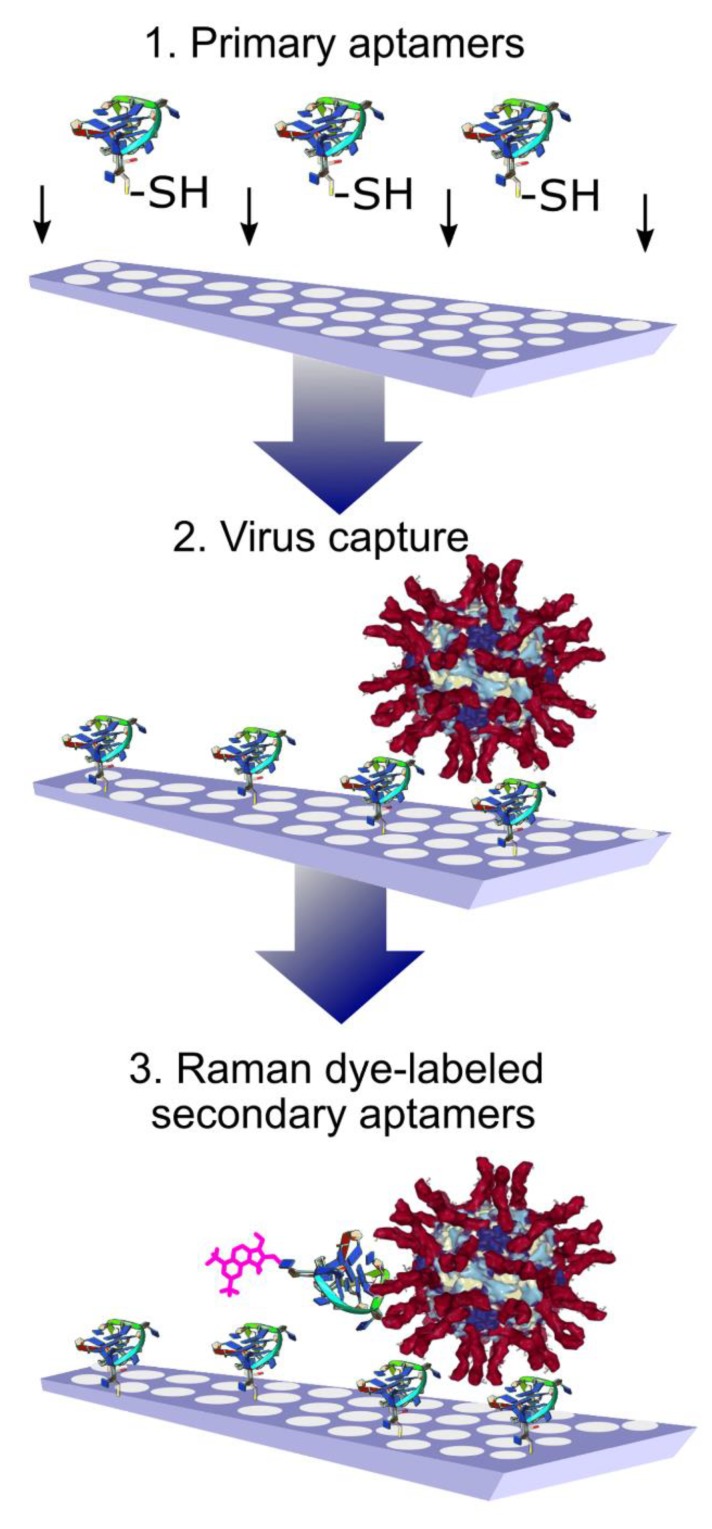
Aptamer-based identification of viral particles on solid substrates. High specificity is achieved due to the presence of a ternary complex with aptamers labeled with Raman-active compounds. Reproduced with permission from [64].

A sandwich-like assay was used for the whole virus capture and identification using aptamers to hemagglutinin of the influenza virus. The primary aptamers were immobilized on the SERS substrates with thiol-groups. After capturing the influenza viral particles, the secondary aptamers labeled with Raman dye were added (Figure 4). The usage of Raman dyes made it possible to perform the assay in biological fluid in a few minutes. A variety of influenza A viral strains were successfully detected [64]. These SERS aptasensors have an obvious benefit in terms of the duration of analysis compared to traditional PCR or even SERS immunosensors with polyclonal antibodies [65].

A similar technique was applied using antibodies instead of aptamers. The immunosensor was developed to detect the hepatitis B virus [66]. Polyclonal antibodies to the hepatitis B virus were immobilized on the surface; then, the viral particles were bound and stained with Au NP modified with monoclonal antibodies to the hepatitis B virus and 4-mercaptobenzoic acid as a reporter molecule. SERS was enhanced with Ag coating on the probes. The detection limit of this technique was 0.5 μg/mL of hepatitis B virus surface antigen, and the test duration was 4 h 20 min.

### 5.4. ASO-Based Test Strips for Viral Genome Detection

Diagnostic test strips based on lateral flow assays are widely used for self-diagnosis and in medical institutions, but in some cases, they have insufficient sensitivity. This problem can be solved by using test strips as biosensors for SERS [67].

Fu et al. proposed test strips using gold nanoparticles with functionalized ASO for the genome of the human immunodeficiency virus [68]. A test strip has a sample area, a conjugation area, a test line, a control line, a nitrocellulose membrane, and an adsorbent. Complexes of streptavidin-biotinylated ASO and streptavidin-biotinylated control DNA were immobilized on the test line and the control line areas, respectively. A sample with the viral genome was applied to the sample area; subsequently, the solution migrated due to the lateral flow through the strip. In the conjugation area, the viral genome hybridized with ASO-modified NP. When the resulting complexes reached the test line, they were captured by immobilized ASO, forming a ternary SERS-active complex. The excess of NP continued to migrate to the control line and was captured by the control DNA probe, which was immobilized on the control line. Both lines were examined by SERS. Under optimized conditions, the detection limit was 0.24 pg/mL, which is lower than that of colorimetric or fluorescent detection methods. These results demonstrate the potential of analyses based on SERS for the quantitative and qualitative identification of pathogens.

Similar tests could be performed without SERS detection, but additional amplification of the viral genome is necessary to increase the sensitivity of the technique. In the strip system, Au NPs were hybridized with the PCR amplified genome of the hepatitis C virus in the test zone; the accumulation of the hybrids results in a red line [69]. Portable SERS equipment could substantially decrease the duration of analyses due to the high sensitivity of the techniques and their applicability to point-of-care services. Ideally, the SERS equipment is to be of the same size as modern individual glucometers.

## 6. Conclusions and Perspectives

Despite the potential power of oligonucleotide-modified SERS substrates as a biosensing tool, there are three main critical issues that need to be solved before widespread application. The first problem is the need to produce inexpensive, chemically stable and reliable SERS substrates with uniformly high enhancement and reproducibility. We have mentioned that ‘classic’ nanomaterials are widely used, providing the sensitivity and stability of SERS-biosensors, for example, metal nanoparticles, metal nanoislands or nanorods on dielectric and other nanostructured materials. SERS enhancement and reproducibility also critically depends on the substrate morphology; therefore, the development of easy and low-cost fabrication methods for biocompatible nanostructures with specific sizes, shapes, alignments, and architectures is still a great challenge. To date, electron beam lithography (EBL) is the most common way to produce highly ordered nanostructured surfaces. However, it is a time-consuming and expensive process that is unable to produce batches of substrates, thereby hampering the mass production of SERS substrates. Photolithography solves these problems and makes possible the production of a large number of substrates in one cycle, thereby reducing the cost of production. It should be noted that photolithography cannot create structural elements with gaps below the diffraction limit (~300 nm). Nevertheless, the transition to longer laser excitation wavelengths (infrared radiation) makes it possible to use photolithographic structures with submicron characteristic sizes (~500 nm) in SERS measurements, with potential applications in biosensing [20].

The controlled creation of metal-dielectric nanostructures plays a key role in the manufacture of reproducible SERS biosensors. The mass production of nanoscale substrates is possible due to modern nanoimprint techniques. A stamp template produced using EBL has been used to create SERS-substrates. One of techniques of direct superplastic nanoimprinting of crystalline metals used a temperature mode well below the melting temperatures of the metals [70]. SERS-biosensors of this type have been already used to detect bacteria and toxins [71,72].

The second issue that may enhance the sensitivity and reproducibility of SERS-based biosensors is the application of statistics methods for automatic analyses of multiparameter spectral data. For example, the projection method on latent structures with linear discriminant analyses makes it possible to create a projection model that distinguishes similar spectra [73]. Other methods, such as partial least squares regression, the variable importance in projection method and the selectivity ratio method are able to identify spectral lines in complex mixtures [74]. The application of the aforementioned or novel statistical methods for the analysis of the spectra of infected biofluids could be a powerful tool to improve the sensitivity of SERS.

As for the specificity of identification, oligonucleotide-based biosensors exhibit great promise. ASO are able to precisely detect the pathogen strain at the genome level, whereas aptamers are useful for the identification of intact virus particles. Chemical synthesis with a wide range of modifications is an advance of oligonucleotides rather than proteins. A comparison of ASO, aptamer and antibody-based biosensors for virus detection is provided in Table 1. The recognition of the whole virus provides lower limits of identification due to higher amounts of viral proteins compared to a single genome copy per virus. Moreover, sandwich-like assays are more robust in the case of whole viruses, as dozens and even hundreds of labeled secondary recognition elements can be absorbed by the virus. The quantity of genome fragments can be easily amplified by PCR; however, this step is time-consuming. Therefore, aptamer-based assays seems to be the most promising and robust approach for express diagnostics. This direction is of emerging interest for further development.

## Figures and Tables

**Table 1:** Comparison between the characteristics of different types of biosensors in the identification of the same type of virus. The indirect SERS-based assays are summarized based on the recognition element: ASO, aptamer or antibody.

Virus	Recognition Element	Biosensor	Target Molecule	Limit of Detection	Time to Result, Min	Refe-rences
Influenza virus	ASO	Solid substrate with immobilized ASO labeled with a dye	Viral RNA	2.7 × 10^−12^ mole per sample (1.6 × 10^6^ viral particles per sample)	480	[59]
	Antibodies	Solid substrate with immobilized polyclonal antibodies + labeled secondary monoclonal antibodies	Viral particles	4.1 × 10^3^ TCID/mL (10 viral particles per sample)	200	[65]
	Aptamers	Solid substrate with immobilized primary aptamers + labeled secondary aptamers	Viral particles	10^−4^ HAU per probe (100 viral particles per sample)	12	[64]
Hepatitis B virus	ASO	Colloid nanoparticles functionalized with labeled ASO for directed aggregation of nanoparticles on solid substrates	Viral DNA	1.4 × 10^−16^ mole (800 viral particles per sample)	240	[55]
	Antibodies	Solid substrate functionalized with primary antibodies + labeled secondary antibodies linked to nanoparticles	Surface antigen (membrane proteins)	0.5 µg/mL (2 × 10^9^ viral particles per sample)	260	[65]

## Abbreviations

SERS	Surface-enhanced Raman spectroscopy
PCR	Polymerase chain reaction
ELISA	Enzyme-linked immunoassay
RS	Raman spectroscopy
LSPR	Localized surface plasmon resonance
NP	Nanoparticle
ASO	Antisense oligonucleotides
PCA	Principal component analysis
